# Ex Vivo Confocal Laser Scanning Microscopy in Rare Skin Diseases

**DOI:** 10.3390/cancers16091713

**Published:** 2024-04-28

**Authors:** Luis Messner, Maximilian Deußing, Michaela Maurer, Lisa Buttgereit, Lara Stärr, Lars E. French, Daniela Hartmann

**Affiliations:** 1 Department of Dermatology and Allergy, LMU University Hospital, LMU Munich, 80337 Munich, Germanydaniela.hartmann@med.uni-muenchen.de (D.H.); 2 Department of Dermatology & Cutaneous Surgery, Miller School of Medicine, University of Miami, Miami, FL 33136, USA

**Keywords:** fluorescence, reflectance confocal microscopy, dermatohistopathology, bedside histology, basal cell carcinoma, squamous cell carcinoma, diagnostic imaging, dermatofibroma protuberans, atypical fibroxanthoma, fibrosarcoma, syphilis, leiomyoma, sarcoma, lymphoma, cutaneous T-cell lymphoma, pseudolymphoma, prurigo nodularis, cylindroma

## Abstract

**Simple Summary:**

This study investigated a new imaging technique called ex vivo confocal microscopy to examine rare skin conditions. By analyzing tissue samples from different skin disorders, we found that this technique could accurately identify unique microscopic features of both common and rare skin diseases. Importantly, examiners with more experience in interpreting these images achieved higher accuracy in diagnosis. This suggests that ex vivo confocal microscopy has the potential to be a valuable tool alongside traditional methods for diagnosing rare skin conditions early and accurately, leading to better treatment outcomes for patients.

**Abstract:**

While ex vivo confocal laser scanning microscopy has previously demonstrated its utility in most common skin diseases, its use in the assessment of dermatological entities with lower incidence remains unexplored in most cases. We therefore aimed to evaluate the diagnostic efficacy of some rare skin tumors as well as a few inflammatory skin diseases, that have not yet been studied in ex vivo confocal laser scanning microscopy. A total of 50 tissue samples comprising 10 healthy controls, 10 basal cell carcinoma, 10 squamous cell carcinoma, and 20 rare skin conditions were imaged using the newest generation ex vivo confocal microscopy (Vivascope 2500 M-G4, Vivascope GmbH, Munich, Germany). Three blinded investigators were asked to identify characteristic features of rare skin disorders and distinguish them from more common skin diseases in the ex vivo confocal microscopy images. Our findings present the capability of ex vivo confocal microscopy to display distinctive morphologic patterns in common and rare skin diseases. As might be expected, we found a strong correlation between imaging experience and diagnostic accuracy. While the imaging inexperienced dermatohistopathologist reached 60% concordance, the imaging-trained dermatologist obtained 88% agreement with dermatohistopathology. The imaging-trained dermatohistopathologist achieved concordance up to 92% with gold-standard dermatohistopathology. This study highlights the potential of ex vivo confocal laser scanning microscopy as a promising adjunct to conventional dermatohistopathology for the early and precise identification of rare dermatological disorders.

## 1. Introduction

Skin diseases encompass a large spectrum of inflammatory and neoplastic diseases, including commonly diagnosed conditions such as basal cell carcinoma (BCC) or squamous cell carcinoma (SCC), as well as a multitude of less common and even rare dermatological disorders. The rising incidence of skin diseases in general, with nearly one in two Europeans experiencing a dermatological condition in the past twelve months, underscores the urgent need for precise diagnostic tools that can enhance the efficiency of finding the correct diagnosis and timely treatment planning [1]. Besides clinical examination, especially in rare skin disorders, dermatohistopathological examination of excised tissue proves to be the gold standard. However, the histological process is often time-consuming and may delay the initiation of appropriate treatments [2,3].

Ex vivo confocal laser scanning microscopy (EVCM) is a promising imaging modality, offering a real-time, high-resolution visualization of skin structures at cellular and subcellular levels within a few minutes. Thanks to the novel digital staining software, the acquired microscopic images show a remarkable resemblance to conventional dermatohistopathologic haematoxylin-eosin (H&E) staining. This improvement makes it even more accessible for trained dermatohistopathologists to use EVCM effectively.

While EVCM has already demonstrated its operational readiness in the examination of healthy skin [4], most common skin tumors [5,6], inflammatory diseases [7], infectious diseases [8], and in combination with fluorescent-labeled antibodies [8,9], its use in the assessment of uncommon and even rare skin diseases remains with exception of a few case reports [10,11] relatively unexplored. Hence, we have chosen the following skin conditions that have not yet undergone comprehensive investigation with EVCM and generally exhibit low incidences: dermatofibrosarcoma protuberans (DFSP) (N = 3), atypical fibroxanthoma (AFX) (N = 3), myxofibrosarcoma, leiomyoma (N = 2), sarcoma, fibrosarcoma, syphilis, lymphoma, cutaneous T-cell lymphoma, pseudolymphoma, prurigo nodularis, cylindroma, undifferentiated SCC, pigmented purpuric dermatosis (Morbus Schamberg) and granulation tissue (Table 1).

Our main task in the study was to determine whether the pattern recognition known from dermatohistopathology was also possible in EVCM in less common or rare diseases that had never been closely studied or described in EVCM yet compared to more common diseases already well studied in EVCM. The second task was to compare the performances of three examiners with different backgrounds, experiences, and areas of expertise. This information is crucial for further use of EVCM for bedside histology, since common diagnoses such as SCC or BCC must be securely distinguished from other rare diseases.

## 2. Materials and Methods

The study was conducted per the Declaration of Helsinki and was approved by the Ludwig Maximilian University Ethics Committee, Munich, Germany (Protocol Nr. 19-150). Each patient gave written informed consent before inclusion in the study.

From November 2020 to April 2023, N = 50 tissue samples were collected from 50 patients enrolled at the Department of Dermatology and Allergy, University Hospital, LMU Munich, Germany. The patient cohort included 10 healthy controls, 10 BCC, 10 SCC, and 20 rare skin diseases that EVCM has not studied in detail until now. Table 1 shows an overview of the investigated diseases and their reported incidence [12,13,14,15,16,17,18,19,20,21,22].

**Table 1:** Overview of investigated diseases and their incidence, n.a. = non applicable.

	Sample Size (N)	Incidence
Healthy skin	10	n.a.
Basal cell carcinoma	10	100–800/100,000 [12]
Squamous cell carcinoma	10	5–500/100,000 [13]
Rare/unstudied skin diseases	20	n.a.
Dermatofibrosacoma protuberans	3	≈1–5/100,000 [14]
Atypical fibrosarcoma	3	≈2.5/100,000 [15]
Myxofibrosarcoma	1	<1/100,000 [16]
Cutaneous leiomyoma	2	<1/100,000 [17]
Pleomorphic dermal sarcoma	1	<1/100,000 [23]
Fibrosarcoma	1	<1/100,000 [24]
Syphilis	1	8.9/100,000 [25]
Primary cutaneous B-cell lymphoma	1	10–15/100,000 [26]
Cutaneous T-cell lymphoma	1	≈0.5–2/100,000 [18]
Cutaneous pseudolymphoma	1	<1/100,000 [19]
Prurigo nodularis	1	≈72/100,000 [20]
Cylindroma	1	<1/100,000 [21]
Undifferentiated SCC	1	n.a.
Morbus Schamberg	1	≈50/100,000 [22]
Granulation tissue	1	n.a.

EVCM examination: Freshly excised tissue was immediately stained and scanned following a standardized protocol: The staining process consists of immersing the probes in Acridine Orange (0.1 mmol/L, Sigma-Aldrich, St. Louis, MO, USA) for 30 s, followed by a 30-s rinse with phosphate-buffered saline (0.1 mmol/L, Dulbecco’s Phosphate Buffered Saline; PBS; pH 7.4, Sigma-Aldrich, St. Louis, MO, USA) in order to remove excess stain. Subsequently, the tissue probes were coated 30 s with citric acid (0.1 mmol/L) for aceto-whitening. Afterward, the tissue probes were positioned on object slides, mounted with sponges and magnets [27], and examined in vertical mode. The parameters for imaging were standardized across all samples to ensure consistency and comparability.

The commercially available EVCM Vivascope 2500 G-4 device (Vivascope, Munich, Germany) is equipped with two different lasers with wavelengths of 488 nm (blue) and 638 nm (red). It examines the samples simultaneously in reflectance mode (RM), fluorescence mode (FM), overlay mode (OM), and digital staining mode (DHE). This study was performed solely in DHE mode, as digital H&E-like images are easier to interpret for EVCM-unexperienced dermatohistopathologists. Further technical details regarding the EVCM, tissue preparation, and examination are provided elsewhere [3,27,28]. Following the EVCM analysis, all samples were fixed in a formaldehyde solution for a gold standard dermatohistopathologcial examination and were independently analyzed in the Dermatohistopathological Department of the Department of Dermatology and Allergy, University Hospital, LMU Munich.

Image evaluation: Overview and detailed DHE EVCM images were presented in a randomized order. Three blinded investigators, an EVCM-trained dermatohistopathologist (D.H.), an EVCM-unexperienced dermatohistopathologist (M.M.), and an EVCM-trained dermatologist with no experience in dermatohistopathology (M.D.), were asked to categorize the images into “rare skin diseases”, BCC, SCC, or healthy control group. Subsequently, the investigators were instructed to analyze the cellular morphology and tissue architecture to describe characteristic morphologic features and patterns of the shown skin diseases.

Comparative Analysis and Dermatohistopathology: In parallel, all skin samples underwent conventional dermatohistopathological examination to establish the gold standard diagnosis. The dermatohistopathological assessments were conducted by independent, experienced dermatohistopathologists following standardized protocols, including the incorporation of immunostaining techniques. Dermatohistopathological results were then compared to the results obtained from EVCM analysis in order to determine the concordance and discrepancy between the two diagnostic modalities. Descriptive statistical calculations were performed using Microsoft Excel 2016 (Microsoft, Redmond, WA, USA).

## 3. Results

EVCM demonstrated high diagnostic efficacy in identifying characteristic features of uncommon and even rare skin diseases. The mean concordance with dermatohistopathology of all three investigators combined was 80%, while the EVCM-trained dermatohistopathologist (D.H.), performed best (46/50 = 92%) compared to the EVCM-trained dermatologist with no experience in dermatohistopathology (M.D.) (44/50 = 88%) and the EVCM-unexperienced dermatohistopathologist (M.M.) (30/50 = 60%).

Table 2 illustrates the performance of each examiner subdivided into four classification groups (BCC, SCC, rare skin diseases, and healthy control). Regarding the evaluation of BCC, the EVCM-trained dermatohistopathologist demonstrated a sensitivity of 0.90, specificity of 0.98, positive predictive value (PPV) of 0.90, and negative predictive value (NPV) of 0.98. Regarding SCC image interpretation, a sensitivity of 0.82, specificity of 0.95, PPV of 0.82, and NPV of 0.95 could be reached. In the classification of rare skin diseases, this investigator achieved a sensitivity of 0.95, specificity of 0.97, PPV of 0.95, and NPV of 0.97, and correctly identified all healthy controls with sensitivity, specificity, PPV, and NPV of 1.00.

The EVCM-trained dermatologist achieved a sensitivity of 0.80 in the identification of SCC, a specificity of 0.95, a PPV of 0.80, and an NPV of 0.95. For the identification of BCC, this examiner saw a sensitivity of 0.73, specificity of 0.95, PPV of 0.80, and NPV of 0.93. The same examiner demonstrated a sensitivity of 0.95, specificity of 0.94, PPV of 0.90, and NPV of 0.97 in the group of rare skin diseases and also correctly identified all healthy controls with sensitivity, specificity, PPV, and NPV of 1.00.

The dermatohistopathologist achieved a sensitivity of 0.60, specificity of 0.93, PPV of 0.67, and NPV of 0.90 and sensitivity of 0.36, specificity of 0.85, PPV of 0.40, and NPV of 0.83 for BCC and SCC, respectively. In the assessment of rare skin diseases, the dermatohistopathologist presented a sensitivity of 0.68, specificity of 0.74, PPV of 0.62, and NPV of 0.79. The healthy controls were identified with a sensitivity of 0.70, specificity of 0.93, PPV of 0.70, and NPV of 0.93.

Notably, the EVCM imaging enabled the visualization of specific cellular and tissue patterns. Referring to conventional dermatohistopathologic descriptions, we observed unique patterns of each skin disease, facilitating their prompt and accurate identification for experienced users (Table 3). In the following, we present the patterns observed in DHE EVCM images and their corresponding skin diseases, complemented by a schematic representation.

**Figure 1:**
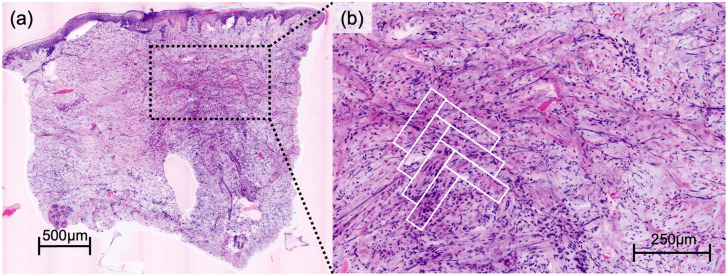
Overview of a digital haematoxylin-eosin image of dermatofibrosarcoma protuberans (**a**) in ex vivo confocal laser scanning microscopy. Detailed view showing interlaced spindle cells forming characteristic herring bone pattern (**b**).

**Figure 2:**
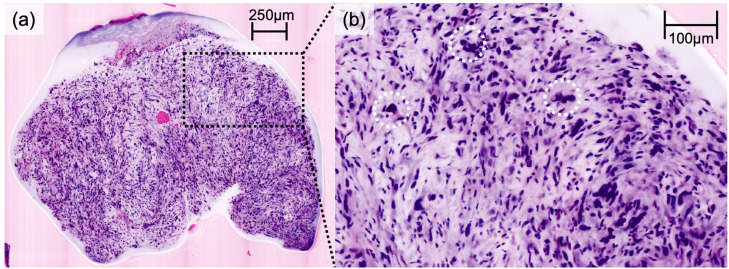
Digital haematoxylin-eosin image of atypical fibrosarcoma (**a**) in ex vivo confocal laser scanning microscopy. Magnification depicting atypical and pleomorphic spindle cells with multinucleated giant cells (highlighted with white circles) (**b**).

**Figure 3:**
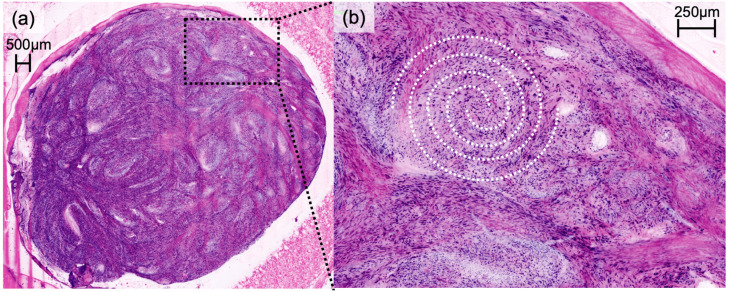
Digital haematoxylin-eosin image of cutaneous leiomyoma (**a**) presenting smooth muscle cells in a whorled pattern (white vortexes) surrounded by interlacing fascicles (**b**) in ex vivo confocal laser scanning microscopy.

**Figure 4:**
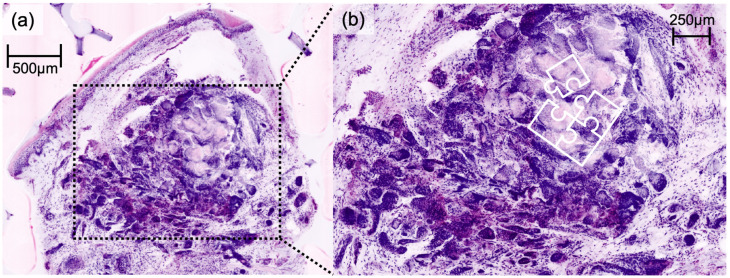
Digital haematoxylin-eosin image of cylindroma (**a**) showing aggregations of basaloid cells forming characteristic jigsaw puzzle-like patterns in ex vivo confocal laser scanning microscopy, also shown in detailed magnification image (**b**).

**Figure 5:**
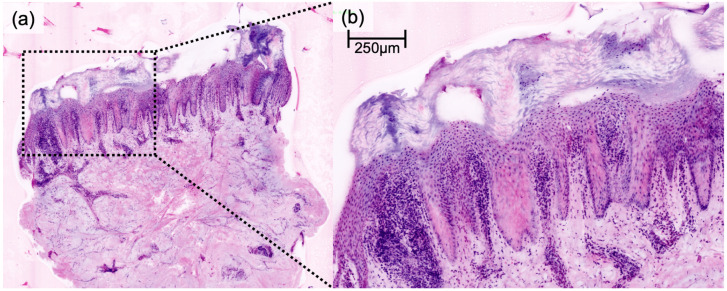
Digital haematoxylin-eosin image of prurigo nodularis lesion (**a**) manifested as hyperkeratosis with underlying dermal fibrosis and linearly arranged collagen fibers in the upper dermis as well as perivascular inflammatory infiltrate (**b**) in ex vivo confocal laser scanning microscopy.

DFSP presented a characteristic honeycomb-like appearance with interlacing spindle cells (Figure 1), while AFX presented with pleomorphic spindle cells and multinucleated giant cells (Figure 2). Irregularly shaped and hypercellular stromal elements characterized fibrosarcoma, while lymphoma displayed dense lymphocytic infiltrates out of proportion with disrupted epidermal architecture (so-called epidermotropism). Leiomyoma presented with smooth muscle cells in a whorled pattern surrounded by interlacing fascicles (Figure 3). Cylindroma (Figure 4) showed aggregations of basaloid cells forming characteristic jigsaw puzzle-like patterns, and prurigo nodularis manifested as hyperkeratotic nodules with underlying dermal fibrosis and perivascular inflammatory infiltrate (Figure 5), among other distinctive features observed in each of these skin diseases.

## 4. Discussion

Our study illustrates the potential of EVCM in the accurate assessment of not only common but also rare skin diseases. It is imperative to highlight the primary objective of EVCM as a tool for pattern recognition, aiming for rapid diagnoses directly at the bedside or in the operation room. Therefore, the unique advantage of EVCM lies in its near real-time visualization capabilities, allowing for rapid identification of characteristic histologic features and patterns, facilitating the decision-making process during surgical procedures. Our results, as seen in Figure 1, Figure 2, Figure 3 and Figure 4, affirm the feasibility of this approach, revealing that the distinctive histologic features, cellular arrangements, specific architectural patterns, and unique cellular morphology specific to each rare skin disease may be readily apparent in the EVCM images.

As might be expected, we found a strong correlation between imaging experience and diagnostic accuracy. Although we hoped to observe greater skill transferability between conventional histology and EVCM, the outcomes exhibited variations in distinguishing between healthy, BCC, SCC, and rare skin diseases and identifying specific patterns depending on the examiner’s training. While the imaging inexperienced dermatohistopathologist reached 60% concordance, the imaging-trained dermatologist obtained 88% agreement with dermatohistopathology. Only imaging-trained dermatohistopathologists achieved a concordance of up to 92% with the gold standard dermatohistopathology.

Not all patterns outlined in this study were universally recognized by all examiners. This is not surprising, given the examiners’ lack of prior exposure to these patterns in EVCM. We hypothesize that their proficiency will notably improve after target-oriented training or a comprehensive study of this manuscript.

While we demonstrate that characteristic patterns of rare skin diseases may be identifiable in EVCM, it is essential to note that larger-scale investigations are imperative to determine key parameters, such as the sensitivity and specificity of the aforementioned features and patterns. Furthermore, the current quality of EVCM images may not consistently match the quality of a conventional histological slide, adding complexity to the analysis. Consequently, we propose that EVCM could serve as a supplementary tool in the diagnostic process of rare skin diseases, contingent upon the reliable identification of distinct features and patterns in skin biopsies, allowing for real-time diagnosis.

Regarding the use of EVCM modes, Vladimirova et al. showed that a variety of dermatohistopathological features could be identified using all four EVCM modes [3]. Each mode specializes in accentuating specific structures or characteristics; FM particularly emphasizes cell nuclei and enhances the contrast of cell structures, while RM is particularly useful for the analysis of matrix structures such as elastic and collagen fibers [3]. Although OM entails the strength of the FM and RM, its appearance differs significantly from conventional histological images, unlike the DHE images [3]. While the relatively new integration of FM, RM, OM, and DHE modes is a powerful diagnostic tool, it requires significant EVCM training for dermatohistopathologists to be proficient in all modes. In our study, we, therefore, focused only on the DHE since the DHE images are easier for EVCM-unexperienced dermatohistopathologists to interpret. However, our results suggest that some EVCM training is necessary for H&E-trained dermatohistopathologists to achieve adequate results. The EVCM-trained dermatohistopathologist and the EVCM-trained dermatologist with no experience in dermatohistopathology successfully identified BCC and SCC, achieving a high level of accuracy in distinguishing them from both healthy samples and rare skin diseases. However, The EVCM-unexperienced dermatohistopathologist did not perform as well despite a significant training in dermatohistopathology. These findings may explain different levels in accuracy using EVCM in the literature [29,30]. Nonetheless, it appears that the DHE mode may be sufficient to identify BCC, SCC, rare skin diseases and healthy skin. Consequently, dermatologists in a clinical setting may be able to identify more complex skin samples that may require a more detailed analysis using all four modes by an EVCM-trained dermatohistopathologist.

A primary limitation of our study, which may have influenced the generalizability of the findings, was the limited sample size due to the rarity of the investigated diseases. Therefore, rare skin diseases warrant further investigation to determine the extent of skin diseases that can be accurately diagnosed using EVCM. Nonetheless, we demonstrated that the few mentions of the studied rare diseases in EVCM literature did not prevent EVCM-trained dermatohistopathologists and EVCM-trained dermatologists from correctly differentiating between rare skin diseases, BCC, SCC, and healthy skin. The next steps should be the study of histological patterns of rare skin diseases in EVCM in a larger setting and the determination of the time needed to train an EVCM-unexperienced dermatohistopathologist. The use of fluorescent-labeled antibodies may further increase the scope [9].

## 5. Conclusions

To conclude, our comprehensive analysis of uncommon and rare skin diseases using EVCM has highlighted its promising role as a rapid and reliable imaging modality in the realm of dermatohistopathology. The high diagnostic accuracy and concordance between the EVCM findings and conventional dermatohistopathological assessments underline the utility of EVCM as an effective supplementary diagnostic tool for distinguishing rare skin diseases from more prevalent conditions. We show that even though EVCM has primarily been designed for fast intraoperative decisions in Mohs surgery, it offers the possibility to recognize and differentiate less common diagnoses in order to maximize patient safety. Nevertheless, it is crucial to ascertain specific training for EVCM-unexperienced dermatohistopathologists and dermatologists to attain proficiency in its use.

## Figures and Tables

**Table 2:** Diagnostic performance metrics of all three investigators divided into four disease classification groups.

	EVCM-Trained Dermatohistopathologist (D.H.)	EVCM-Trained Dermatologist with No Experience in Dermatohistopathology (M.D.)	EVCM-Unexperienced Dermatohistopathologist (M.M.)
BCC (n = 10)			
Sensitivity	0.90	0.80	0.60
Specificity	0.98	0.95	0.93
PPV	0.90	0.80	0.67
NPV	0.98	0.95	0.90
SCC (n = 10)			
Sensitivity	0.82	0.73	0.36
Specificity	0.95	0.95	0.85
PPV	0.82	0.80	0.40
NPV	0.95	0.93	0.83
Rare skin diseases (n = 20)			
Sensitivity	0.95	0.95	0.68
Specificity	0.97	0.94	0.74
PPV	0.95	0.90	0.62
NPV	0.97	0.97	0.79
Healthy control (n = 10)			
Sensitivity	1.00	1.00	0.70
Specificity	1.00	1.00	0.93
PPV	1.00	1.00	0.70
NPV	1.00	1.00	0.93

**Table 3:** Characteristic morphologic features observed in rare skin diseases using EVCM.

Diagnosis	Dermatohistopathological Features
Dermatofibrosarcoma protuberans	Spindle cells with a storiform and/or herringbone pattern
Atypical fibroxanthoma	Bizarre and atypical cells with marked pleomorphism in size and shape
Myxofibrosarcoma	Lobulated tumor with multinodular growth and incomplete fibrous septa
Cutaneous leiomyoma	Smooth muscle cells with whorled pattern and interlacing fascicles
Cutaneous T-cell lymphoma	Intraepidermal lymphocytes with epidermotropism ± Pautrier microabscesses
Cutaneous pseudolymphoma	Dermal lymphocytic infiltrate forming follicles
Prurigo nodularis	Compact orthokeratosis with focal parakeratosis and perivascular mixed inflammatory infiltrate
Cylindroma	Multiple irregular tumor islands distributed in a jigsaw pattern
Undifferentiated SCC	Nests and sheets of tumor cells, necrosis, lack of signs of differentiation
Morbus Schamberg	Epidermal spongiosis, hemosiderin deposition, and variable pigmentation incontinence
Granulation tissue	Plump fibroblasts, reactive endothelial cells, and mixed inflammatory infiltrates

## Data Availability

The data presented in this study are available on request from the corresponding author. The data are not publicly available due to ethnical and privacy restrictions.
